# COVID‐19 pandemic impact on blood donations and discards from screening tests for transfusion‐transmitted infections in a Brazil Brazilian metropolitan area

**DOI:** 10.1111/tme.13159

**Published:** 2025-07-07

**Authors:** Laiane da Silva Santos, Sérgio Eduardo Soares Fernandes, Flávio Ferreira Pontes Amorim, Anna Luiza Oliveira Sant'Anna, Felipe Ferreira Pontes Amorim, Fábio Ferreira Amorim

**Affiliations:** ^1^ Graduate Program of Health Sciences Escola Superior de Ciências da Saúde Brasília Brazil; ^2^ Institutional Research and Development Committee Fundação Hemocentro de Brasília Brasília Brazil; ^3^ Graduate Program of Health Sciences Universidade de Brasília Brasília Brazil; ^4^ Medical School Universidade Católica de Brasília Brasília Brazil; ^5^ Medical School UNICEPLAC Centro Universitário do Planalto Central Apparecido dos Santos Brasília Brazil

**Keywords:** blood donation, blood safety, blood transfusion, blood‐borne infections, blood‐borne pathogens, COVID‐19, motivation

## Abstract

**Background:**

The COVID‐19 pandemic posed a worldwide challenge for blood services. We aimed to evaluate the impact of the COVID‐19 pandemic on blood donations and blood discards resulting from screening tests for transfused‐transmitted infections (TTIs) in a Brazilian metropolitan.

**Methods:**

Time‐series cohort study including data of all blood donors from January 2018 to December 2021 at the Brasília Blood Center Foundation, Federal District, Brazil. The causal impact analysis was used to evaluate the impact of COVID‐19 on blood donations, and a propensity score matching was used to evaluate the effect of the COVID‐19 pandemic on the seroprevalence of TTIs.

**Results:**

There were 205 965 blood donations during the study period. The blood donations significantly reduced soon after the onset of the COVID‐19 pandemic in Brasilia, Brazil, in March 2020 until August 2020 (absolute effect per week: −2952; 95% CI: −4627 to −1355). However, from September 2020 to December 2021, blood donations had returned to the levels foreseen by the time‐series model. Compared to the pre‐COVID‐19 period, the period between September 2020 and December 2021 was associated with a decrease of at least one reactive or indeterminate screening test for TTI (OR: 0.753, 95% CI: 0.665–0.854, *p* <0.001).

**Conclusion:**

There was a substantial decrease in blood donations soon after COVID‐19 onset in Brazil. However, within a few months, the donation levels had returned to those projected by the model, possibly due to measures implemented by the blood centre for blood donations. The seroprevalence of TTIs decreased during the COVID‐19 pandemic.

## 1 INTRODUCTION

The COVID‐19 pandemic posed a severe challenge to healthcare systems, rapidly infecting a vast population and causing high morbidity and mortality before vaccines became available. Declared in March 2020, the pandemic resulted in over 7 million confirmed deaths globally, with estimates exceeding 15 million due to underreporting.^1^, ^2^, ^3^, ^4^, ^5^ Beyond its direct health impact, it triggered economic crises, educational disruptions, and a rise in mental health issues. In this context, various countries implemented measures, such as healthcare service reorganisation, social distancing, and lockdowns, to curb the viral spread and reduce healthcare strain. In May 2023, the WHO declared the end of the global emergency, yet the pandemic's effects on healthcare services may serve as a foundation for future pandemic preparedness.^1^, ^2^, ^3^, ^4^

Annually, approximately 118.5 million blood donations are collected worldwide to support diverse clinical needs.^6^ To meet this demand, blood centres faced significant challenges during the early stages of the pandemic, including a decline in donations and reported shortages in blood supply worldwide.^4^, ^7^, ^8^, ^9^, ^10^, ^11^, ^12^, ^13^ In this context, a systematic review and meta‐analysis of 38 articles found a mean 38% decrease in blood donations, reaching 67% in some regions, primarily due to fear of infection and social restrictions.^9^ A study in Africa comparing the first five months of 2019 and 2020 reported that blood donations declined in 32 countries but increased in five during the COVID‐19 pandemic.^11^

Brazil emerged as one of the most profoundly impacted nations by COVID‐19.^6^, ^14^ Following the first case of COVID‐19 reported on February 26, 2020, in São Paulo city, the contagion initially permeated Brazil's foremost metropolitan areas in the Southeast.^6^, ^14^, ^15^ Its rapid dissemination crossed several regions, starting in the North and Northeast and extending to the Midwest and South. The disease's epicentre underwent dynamic shifts during the pandemic, resulting in three consequential waves of infections and fatalities from March 2020 to January 2022. As the pandemic unfolded, by the end of 2022, Brazil had tallied a staggering 36.3 million confirmed COVID‐19 cases, constituting approximately 5% of the caseload worldwide. Furthermore, it garnered the unenviable distinction of ranking second in global mortality, with about 10% of the worldwide tally of COVID‐related deaths.^6^, ^15^ Therefore, healthcare services in Brazil, including blood centres, are probably among the most impacted by the pandemic in the world. In this context, a previous Brazilian study, analysing data from the initial months of the pandemic (March–June 2020) in blood banks in the state of Minas Gerais, showed a 17% average reduction in donor attendance after the onset of the COVID‐19 pandemic, compared to a historical series from 2016 to 2019, with the most significant drop (19%) occurring in April 2020.^12^

Safe and sufficient blood supply is fundamental to every country's national healthcare policy and infrastructure.^6^, ^9^ In Brazil, more than 3 million blood donations are collected annually based on non‐remunerated replacement or voluntary donors, a practice instituted since the prohibition of paid blood donations in the early 1980s. Besides, 70% of blood donations are sourced through public blood centers.^16^, ^17^, ^18^, ^19^ To ensure blood safety, all donations should be screened for infections before use. According to the World Health Organisation, screening for human immunodeficiency virus (HIV), hepatitis B virus (HBV), hepatitis C virus (HCV), and syphilis should be mandatory.^6^ In this context, all Brazilian blood centers are required to screen for syphilis, Chagas disease, HBV, HCV, HIV, and human T‐cell lymphotropic virus (HTLV). Furthermore, malaria testing is incorporated in regions where malaria is endemic. When any tests for these infections are reactive or inconclusive, the blood donation should be discarded.^16^, ^17^, ^18^, ^19^

Furthermore, a noteworthy consequence of the COVID‐19 pandemic was a substantial drop in blood collection from voluntary donors, widely regarded as the cornerstone of a secure blood supply.^20^, ^21^, ^22^ This aspect could have influenced blood safety concerning transfusion‐transmitted infections (TTIs).^12^ However, few studies evaluated the trends of TTIs during the pandemic. In this context, a study conducted in the United States observed a decrease in the seroprevalence of HIV and HCV in blood donations during COVID‐19.^13^ Conversely, a study conducted in India reported an increase in the seroprevalence of HIV, HBV surface antigen (HBsAg), HCV, and syphilis during the COVID‐19 pandemic.^22^

Evaluating blood supply and safety during the COVID‐19 pandemic provides valuable insights for blood services and health authorities in developing effective emergency response plans for future crises. This study primarily investigates the impact of the COVID‐19 pandemic on blood donations at a blood centre in a Brazilian metropolitan area using causal impact analysis (CIA). This method enhances traditional statistical approaches by inferring causality rather than mere correlation, constructing a counterfactual scenario to estimate how blood donations would have evolved without the pandemic, and incorporating Bayesian structural time series models to account for trends, seasonality, and fluctuations in donation patterns over time, ensuring a more precise and nuanced analysis.^23^, ^24^ Additionally, the study examines the pandemic's effect on blood discards due to transfusion‐transmitted infections (TTIs), applying the propensity score method (PSM) to enhance the accuracy of comparisons.

## 2 METHODS

### 2.1 Study design

A retrospective time‐series cohort study including all consecutive blood donors from January 2018 to December 2021 at the Brasília Blood Center Foundation (BBCF), Federal District (FD), Brazil. Data were obtained from the electronic medical record and the BBCF database of blood donations.

The study was approved by the institutional review board of the Education and Research Foundation of Health Sciences, Brasília, Federal District, Brazil (number 4659322), with a waiver of informed consent. The study was conducted according to the Declaration of Helsinki. Because this study had no specific intervention and used only anonymised medical record data that generated results in an aggregate manner that did not allow the identification of participants, written consent was unnecessary.

### 2.2 Settings and participants

The FD is a metropolitan area with 2 469 489 inhabitants, including Brasilia, Brazil's capital. The state government established the first lockdown on March 18, 2020, when there were 36 confirmed cases. The lockdown measures included suspending non‐essential activities and closure of gyms, schools, and shopping centres, restricting people's movement, and curbing the transmission of the virus. Thus, the pre‐COVID‐19 onset period was defined as January 2018 to February 2020, and the post‐COVID‐19 onset period was defined as March 2020 to December 2021.

The study included all blood donors at the BBCF from January 2018 to December 2021. No exclusion criteria were applied. The BBCF is the unique blood centre responsible for collecting, testing, and processing all blood donations within the FD public healthcare system, including 16 public hospitals.

Following the BBCF's standardised eligibility criteria for blood donation, all donors are unpaid, aged 16 years or older, and must weigh at least 50 kg. Prospective donors underwent a rigorous pre‐donation interview to assess their suitability. All blood units were subjected to comprehensive screening for:

- HBV: HBV core antigen (anti‐HBc), HBsAg, and nucleic acid testing (NAT) for HBV (NAT‐HBV).
- HCV: anti‐HCV and NAT for HCV (NAT‐HCV).
- HIV 1/2: anti‐HIV 1/2 and NAT for HIV (NAT‐HIV).
- HTLV 1/2: anti‐HTLV 1/2.
- Chagas disease: anti‐*Trypanosoma cruzi* antibodies.
- Syphilis: non‐treponemal antibodies.

### 2.3 Data collection

The variables collected were age, sex, city of residence, blood donation date, and serological and RNA/DNA screening test results.

The chemiluminescence method using the automated Architect i2000SR (Abbott Laboratories, Abbott Park, Illinois, USA) was used to perform all serological tests for infectious diseases in a single‐sample testing for each donated blood. The NAT (Bio‐Manguinhos/Fiocruz, Rio de Janeiro, Brazil) underwent 50 cycles and a sensitivity value of 100 IU/mL for HIV and HCV, 50 IU/mL for HBV in single‐sample testing, 600 IU/mL for HIV and HCV, and 300 IU/mL for HBV in pool‐sample testing.

Blood donation was discarded when at least one serological screening test for TTI was reactive or inconclusive and/or NAT detected HBV, HCV, and/or HIV.

Blood donors with a reactive screening test for TTIs were recalled for confirmatory laboratory testing, which included:

- HBV: Repeating the serological screening test, along with NAT‐HBV and anti‐HBc IgG and IgM tests.
- HCV: NAT‐HCV and Western Blot for HCV.
- HIV: NAT‐HIV and immunoblot for anti‐HIV antibodies.
- HTLV 1/2: Immunoblot for anti‐HTLV 1/2 antibodies.
- Chagas disease: Indirect hemagglutination test (IHA) for anti‐Trypanosoma cruzi antibodies.
- Syphilis: Venereal Disease Research Laboratory (VDRL) test.

### 2.4 Statistical analysis

Statistical analyses were performed using the IBM Statistical Package for the Social Sciences version 20.0 for Mac, Jamovi 2.3.24 (https://www.jamovi.org), and statistical software R version 4.2.3 (https://www.r-project.org/).

The distribution and normality of variables were analysed using the Shapiro–Wilk test. Quantitative data are expressed as mean ± standard deviation (SD), or median and interquartile range (IQR25%–75%), and categorical variables are expressed as numbers and percentages (%).

#### 2.4.1 Blood donations

First, blood donations for each epidemiological week were calculated between January 2018 and December 2021. Then, a causal impact analysis (CIA) was performed using the Causal Impact R package to estimate the causal effect of COVID‐19 on the time series of blood donations that applies a Bayesian structural time series model with predictor data sets to determine the likely trajectory of a trend line had a particular event not occurred (the COVID‐19 pandemic) and then calculated the difference between that projected counterfactual trend line (if COVID‐19 pandemic had not occurred) and the real data line (after the COVID‐19 onset).^23^, ^24^ As a control variable in the model, the CIA of the COVID‐19 pandemic on blood donations was adjusted for the estimated population of the FD on each epidemiological week.

#### 2.4.2 Blood donation discard resulting from screening tests for TTIs

A propensity score matching was used to evaluate the effect of the COVID‐19 pandemic on blood donation discards resulting from screening tests for TTIs.

First, the Cochran‐Armitage test for trend in proportions was performed to compare the blood donation discards due to at least one reactive or indeterminate screening test for TTI between January 2018 and February 2020, March 2020 and August 2020, and September 2020 and December 2021.

Second, a univariate analysis was performed using Student's *t*‐test or Mann–Whitney test to evaluate quantitative variables associated with blood donation discard due to at least one reactive or indeterminate screening test for TTI, as appropriate, and Pearson's chi‐square test was used as necessary for categorical variables.

Subsequently, a multivariate analysis was performed using binary logistic regression analysis with the enter method to assess independent factors, including non‐collinear variables associated with the outcome with a *p*‐value <0.05 in the univariate analysis and the confounding factors according to previous knowledge with a *p*‐value <0.20 in the univariate analysis. Non‐collinearity was accepted when the tolerance was higher than 0.10 and the variance inflation factor (VIF) was lower than 10.0. The odds ratio (OR) expressed the results with their respective 95% confidence interval (95% CI).

Finally, a propensity score matching to evaluate the effect of the COVID‐19 pandemic on blood donation discards resulting from screening tests for TTIs was performed, adjusting for factors independently associated with at least one reactive or indeterminate screening test in the logistic regression analysis, using the Easy R (EZR) software version 1.54 (Saitama Medical Center, Jichi Medical University, Japan) with a 1:1 pair‐matching ratio without replacement on the logit of the propensity score applying a calliper of 0.2 widths. The OR and 95% CI of the COVID‐19 effect on blood donation discards resulting from screening tests for TTIs between March 2020 and August 2020 and September 2020 and December 2021 were calculated in the sample after matching and are shown as a forest plot.

Statistical significance was set at a two‐sided *p*‐value ≤0.05.

## 3 RESULTS

Between March 2018 and December 2021, 205 965 blood donations were performed at the BBCF. The mean age was 33.2 ± 10.8 years, and 84 409 (41.0%) were female. A total of 2485 (1.2%) blood donations were discarded due to reactive or indeterminate screening tests for TTIs: 2378 (1.2%) had one reactive or indeterminate screening test for only one TTI (single agent), and 107 (0.1%) had reactive or indeterminate tests for more than one TTI (mixed agents). Specifically, 1015 (0.5%) donations tested reactive or indeterminate for syphilis, 817 (0.4%) for HBV, 410 (0.2%) for HCV, 323 (0.2%) for HTLV 1/2, 20 (<0.1%) for HIV 1/2, and 11 (<0.1%) for Chagas disease, Table 1.

**TABLE 1:** Characteristics of blood donors and discards resulting from screening tests for transfusion‐transmitted infections from January 2018 to December 2021 (*n* = 205 965).

Age, years	
Mean (SD)	33.2 (10.8)
Median (IQR25%–75%)	32.0 (24.0–40.0)
Female, *n* (%)	84 409 (41.0)
Residence in the Federal District, *n* (%)	181 029 (87.9)
Year of blood donation, *n* (%)	
2018	51 282 (24.9)
2019	52 995 (25.7)
2020	50 369 (24.5)
2021	51 139 (24.9)
Reactive or indeterminate screening test for TTI, *n* (%)	
At least one TTI	2485 (1.2)
Single agent (one TTI)	2378 (1.2)
Mixed agent (more than one TTI)	107 (0.1)
Reactive or indeterminate screening test by TTI, *n* (%)	
Syphilis	1015 (0.5)
HBV	817 (0.4)
HCV	410 (0.2)
HTLV 1/2	323 (0.2)
HIV 1/2	20 (<0.1)
Chagas disease	11 (<0.1)

Abbreviations: HBV, hepatitis B virus; HCV, hepatitis C virus; HIV 1/2, human immunodeficiency virus types I and II; HTLV 1/2, human T‐cell lymphotropic virus types I and II; IQR25%–75%, interquartile range 25%–75%; SD, standard deviation.

**FIGURE 1:**
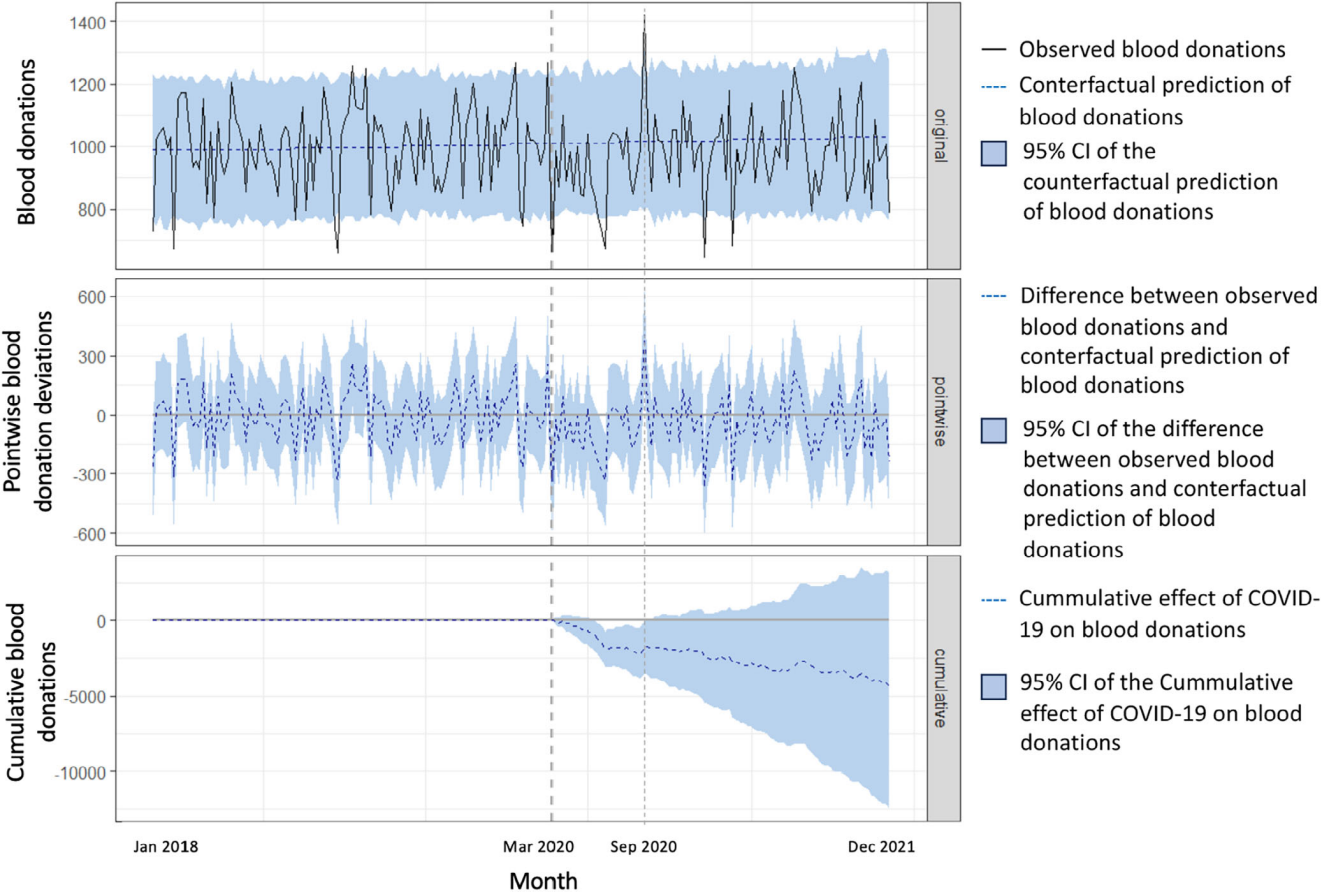
Causal impact analysis of the COVID‐19 pandemic on blood donations performed in the Federal District public health system adjusted to the Federal District population using data from March 2018 to December 2021. The first panel shows the observed blood donations (solid black line), the counterfactual prediction if COVID‐19 had not occurred adjusted to the Federal District population over time (dotted blue line), and the 95% confidence interval (95% CI) of the counterfactual prediction (shaded region). The second panel shows the pointwise causal effect of COVID‐19 on blood donations adjusted for the Federal District population over time. These components represent the difference between observed blood donations and the counterfactual prediction of blood donations adjusted for the Federal District population over time (dotted blue line), along with the 95% CI of this difference (shaded region). The third panel shows the pointwise contributions from the second panel, resulting in a plot of the cumulative effect of COVID‐19 on blood donations over time (dotted blue line), along with the 95% CI of the cumulative effect (shaded region).

Figure 1 and (Table S1) show the CIA of the COVID‐19 pandemic on blood donations in the FD public health system adjusted to the FD population over the study period. The blood donations significantly decreased soon after the COVID‐19 pandemic onset until August 2020. Between the first epidemiological week starting in March 2020 and the last epidemiological week starting in August 2020, there were 23 764 blood donations, while 26 716 (95% CI: 25119–28 391) blood donations would have been expected if the COVID‐19 pandemic had not occurred, with an absolute effect of −2952 (95% CI: −4627 to −1355) blood donations per epidemiological week and a relative effect of −11.0% (95% CI: −16.3% to −5.4%). From September 2020 until the end of the follow‐up period, the cumulative effect of COVID‐19 on blood donations consistently remained within the range predicted by the counterfactual model, indicating no significant deviation from the expected values as if the COVID‐19 pandemic had not occurred. Between the first epidemiological week beginning in September 2020 and the last epidemiological week starting in December 2021, there were 69 381 blood donations, while 68 142 (95% CI: 62638–73 814) blood donations would have been expected if the COVID‐19 pandemic had not occurred, with an absolute effect of 1239 (95% CI: −4433 to 6743) blood donations per epidemiological week and a relative effect of 1.8% (95% CI: −6.0% to 10.8%). For the entire period from the first epidemiological week beginning in March 2020 to the last epidemiological week beginning in December 2021, there were 93 145 blood donations, while 96 817 (95% CI: 89343 to 105 014) blood donations would have been expected if the COVID‐19 pandemic had not occurred, with an absolute effect of −3672 (95% CI: −11 869 to 3802) blood donations per epidemiological week and a relative effect of −3.8% (95% CI: −11.3% to 4.2%).

Table 2 presents the Cochran‐Armitage test for the trend in proportions of blood discards resulting from screening tests for TTIs across different blood donation periods. There was a significant decrease in blood discards due to reactive or indeterminate screening tests for TTIs in the post‐COVID‐19 onset periods (March 2020 to December 2021 and September 2020 and December 2021) compared to the pre‐COVID‐19 period (January 2018 to February 2021), *p* <0.001. Among the TTIs, reactive or indeterminate screening tests decreased significantly for syphilis (*p* < 0.001), HBV (*p* < 0.001), and HTLV 1/2 (p < 0.001).

**TABLE 2:** Cochran‐Armitage test for trend in proportions of blood discards resulting from reactive or indeterminate screening tests for transfusion‐transmitted infections (TTIs) by the period of blood donation (*n* = 205 965).

Blood discard resulting from screening tests for TTIs (at least one reactive or indeterminate screening test for TTI)				
Period	Reactive or indeterminate screening test		% of reactive	*p*‐Value
	Yes	No		
Jan 2018–Feb 2020	1508	111 188	1.3	<0.001
Mar 2020–Aug 2020	291	24 402	1.2	
Sep 2020–Dec 2021	686	67 890	1.0	

Screening test for Syphilis				
Period	Reactive or indeterminate screening test		% of reactive	*p*‐Value
	Yes	No		
Jan 2018–Feb 2020	601	112 095	0.5	<0.001
Mar 2020–Aug 2020	139	24 554	0.6	
Sep 2020–Dec 2021	275	68 301	0.4	

Screening test for HBV				
Period	Reactive or indeterminate screening test		% of reactive	*p*‐value
	Yes	No		
Jan 2018–Feb 2020	504	112 192	0.4	<0.001
Mar 2020–Aug 2020	84	24 609	0.3	
Sep 2020–Dec 2021	229	68 347	0.3	

Screening test for HCV				
Period	Reactive or indeterminate screening test		% of reactive	*p*‐value
	Yes	No		
Jan 2018–Feb 2020	241	112 455	0.2	0.092
Mar 2020–Aug 2020	47	24 646	0.2	
Sep 2020–Dec 2021	122	68 454	0.2	

Screening test for HTLV 1/2				
Period	Reactive or indeterminate screening test		% of reactive	*p*‐value
	Yes	No		
Jan 2018–Feb 2020	206	112 490	0.2	0.001
Mar 2020–Aug 2020	35	24 658	0.1	
Sep 2020–Dec 2021	82	68 494	0.1	

Screening test for HIV 1/2				
Period	Reactive or indeterminate screening test		% of reactive	*p*‐value
	Yes	No		
Jan 2018–Feb 2020	9	112 687	<0.1	0.124
Mar 2020–Aug 2020	0	24 687	0.0	
Sep 2020–Dec 2021	11	68 565	0.1	

Screening test for Chagas disease				
Period	Reactive or indeterminate screening test		% of reactive	*p*‐value
	Yes	No		
Jan 2018–Feb 2020	9	112 687	<0.1	0.125
Mar 2020–Aug 2020	0	24 693	0.0	
Sep 2020–Dec 2021	2	68 574	<0.1	

Abbreviations: HBV, hepatitis B virus; HCV, hepatitis C virus; HIV 1/2, human immunodeficiency virus types I and II; HTLV 1/2, human T‐cell lymphotropic virus types I and II; IQR%25–75%, interquartile range 25%–75%; SD, standard deviation.

This decline in blood discards due to reactive or indeterminate screening tests for TTIs during the COVID‐19 pandemic was observed for single agent and mixed agents (Table S2).

Table 3 shows the confirmatory test results among blood donors with a reactive screening test for transfusion‐transmitted infections (TTIs) across different blood donation periods. A significant decrease in positive/detectable confirmatory test results for syphilis (*p* < 0.001) and HIV (*p* = 0.028) was observed between March 2020 and August 2020 compared to the pre‐COVID‐19 period. However, the positivity rate for syphilis increased between September 2020 and December 2021. Conversely, positive/detectable confirmatory test results for HCV (*p* < 0.001) and HTLV 1/2 (*p* < 0.001) showed an upward trend following the onset of the COVID‐19 pandemic compared to the pre‐pandemic period. Nonetheless, these findings should be interpreted cautiously due to significant differences in the number of individuals who returned for confirmatory testing across the studied periods.

**TABLE 3:** Confirmatory test results among blood donors with a reactive screening test for transfusion‐transmitted infections (TTIs) by the period of blood donation (*n* = 2485).

Syphilis					
Result	Total (*n* = 1015)	Period			*p*‐value
		Jan 2018– Feb 2020 (*n* = 601)	Mar 2020–Aug 2020 (*n* = 139)	Sep 2020–Dec 2021 (*n* = 275)	
Positive/detected, *n* (%)	460 (45.3)	248 (41.3)	52 (37.4)	160 (58.2)	<0.001
Indeterminate, *n* (%)	2 (0.2)	2 (0.3)	0 (0.0)	0 (0.0)	
Negative/non‐detected, *n* (%)	537 (52.9)	344 (57.2)	84 (60.4)	109 (39.6)	
Did not performed, *n* (%)	16 (1.6)	7 (1.2)	3 (2.2)	6 (2.2)	

HBV					
Result	Total (*n* = 817)	Period			*p*‐value
		Jan 2018–Feb 2020 (*n* = 504)	Mar 2020–Aug 2020 (*n* = 84)	Sep 2020–Dec 2021 (*n* = 229)	
Positive/detected, *n* (%)	46 (5.6)	32 (6.3)	3 (3.6)	11 (4.8)	0.230
Indeterminate, *n* (%)	11 (1.3)	10 (2.0)	0 (0.0)	1 (0.4)	
Negative/non‐detected, *n* (%)	741 (90.7)	449 (89.1)	81 (96.4)	211 (92.1)	
Did not performed, *n* (%)	19 (2.3)	13 (2.6)	0 (0.0)	6 (2.6)	

HCV					
Result	Total (*n* = 410)	Period			*p*‐value
		Jan 2018– Feb 2020 (*n* = 241)	Mar 2020– Aug 2020 (*n* = 47)	Sep 2020–Dec 2021 (*n* = 122)	
Positive/detected, *n* (%)	27 (6.6)	12 (5.0)	7 (14.9)	8 (6.6)	<0.001
Indeterminate, *n* (%)	151 (36.8)	97 (40.2)	17 (36.2)	37 (30.3)	
Negative/non‐detected, *n* (%)	106 (25.9)	19 (7.9)	20 (42.6)	67 (54.9)	
Did not performed, *n* (%)	126 (30.7)	113 (46.9)	3 (6.4)	10 (8.2)	

HTLV 1/2					
Result	Total (*n* = 323)	Period			*p*‐value
		Jan 2018–Feb 2020 (*n* = 206)	Mar 2020–Aug 2020 (*n* = 35)	Sep 2020–Dec 2021 (*n* = 82)	
Positive/detected, *n* (%)	44 (13.6)	19 (9.2)	8 (22.9)	17 (20.7)	<0.001
Indeterminate, *n* (%)	28 (8.7)	13 (6.3)	6 (17.1)	9 (11.0)	
Negative/non‐detected, *n* (%)	191 (59.1)	121 (58.7)	19 (54.3)	51 (62.2)	
Did not performed, *n* (%)	60 (18.6)	53 (25.7)	2 (5.7)	5 (6.1)	

HIV 1/2					
Result	Total (n = 20)	Period			*p*‐value
		Jan 2018–Feb 2020 (*n* = 9)	Mar 2020–Aug 2020 (*n* = 0)	Sep 2020–Dec 2021 (*n* = 11)	
Positive/detected, *n* (%)	4 (20.0)	3 (33.3)	0 (0.0)	1 (9.1)	0.028
Indeterminate, *n* (%)	0 (0.0)	0 (0.0)	0 (0.0)	0 (0.0)	
Negative/non‐detected, *n* (%)	5 (25.0)	4 (44.4)	0 (0.0)	1 (9.1)	
Did not performed, *n* (%)	11 (55.0)	2 (22.2)	0 (0.0)	9 (81.8)	

Chagas disease					
Result	Total (*n* = 11)	Period			*p*‐value
		Jan 2018–Feb 2020 (*n* = 9)	Mar 2020– Aug 2020 (*n* = 0)	Sep 2020–Dec 2021 (*n* = 2)	
Positive/detected, *n* (%)	7 (63.6)	5 (55.6)	0 (0.0)	2 (100.0)	0.237
Indeterminate, *n* (%)	0 (0.0)	0 (0.0)	0 (0.0)	0 (0.0)	
Negative/non‐detected, *n* (%)	0 (0.0)	0 (0.0)	0 (0.0)	0 (0.0)	
Did not performed, *n* (%)	4 (36.4)	4 (44.4)	0 (0.0)	0 (0.0)	

Abbreviations: HBV, hepatitis B virus; HCV, hepatitis C virus; HIV 1/2, human immunodeficiency virus types I and II; HTLV 1/2, human T‐cell lymphotropic virus types I and II; IQR%25–75%, interquartile range 25%–75%; SD, standard deviation.

In the univariate analysis, age, female sex, and residence in FD were also associated with at least one reactive or indeterminate screening test for TTIs, Table 4.

**TABLE 4:** Univariate analysis of factors associated with blood discard (at least one reactive or indeterminate screening test for at transfusion‐transmitted infections).

Total			
Variable	Reactive or indeterminate screening test		*p*‐value
	Yes (*n* = 2485)	No (*n* = 203 480)	
Age, years, mean (SD)	35.6 (12.1)	33.1 (10.8)	<0.001
Female, *n* (%)	1212 (48.8)	89 266 (43.9)	<0.001
Residence in the FD, *n* (%)	2063 (83.0)	178 968 (88.0)	<0.001

Abbreviations: FD: federal district; IQR25%–75%, interquartile range 25%–75%; SD, standard deviation.

Table 5 shows the multivariate analysis of variables associated with at least one reactive or indeterminate screening test for TTI. The period of March 2020 to August 2020 (OR: 0.861, 95% CI: 0.758–0.976, *p* = 0.020) and September 2020 to December 2021 (OR: 0.726, 95% CI: 0.663–0.795, *p* < 0.001) were independently associated with a decrease in at least one reactive or indeterminate screening test for TTI compared to pre‐COVID‐19, after adjustment for age, sex, and residence in FD.

**TABLE 5:** Multivariate analysis of factors associated with blood discard (at least one reactive or indeterminate screening test for at transfusion‐transmitted infections).

Variable	OR (95% CI)	*p*‐value	VIF	Tolerance
Age (per year)	1.023 (1.019–1.026)	<0.001	1.03	0.975
Female	1.328 (1.226–1.439)	<0.001	1.02	0.977
Residence in the FD	0.654 (0.589–0.727)	<0.001	1.00	0.999
Period			1.00	0.998
Mar 2020–Aug 2020^a^	0.861 (0.758–0.976)	0.020		
Sep 2020–Dec 2021^a^	0.726 (0.663–0.795)	<0.001		

Abbreviations: 95% CI, 95%: confidence interval; OR, odds ratio; VIF, variance inflation factor.

^a^ The reference group is the Jan 2018–Feb 2020 period.

**FIGURE 2:**
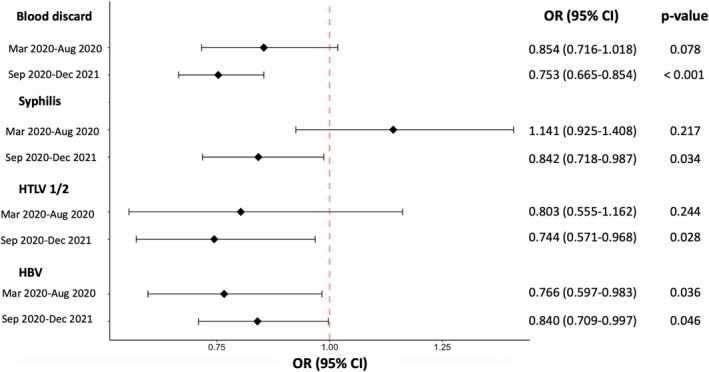
Effect of the post‐COVID‐19 onset period on blood discards resulting from screening tests for transfusion‐transmitted infections after propensity score matching adjusted for age, sex, and residence in the Federal District. 95% CI: 95%, confidence interval; HBV, hepatitis B virus; HTLV 1/2, human T‐cell lymphotropic virus types I and II; OR, odds ratio.

Figure 2 shows the effect of COVID‐19 on blood donation discards resulting from screening tests for TTIs after propensity score matching adjusted for age, sex, and residence in the FD. Compared to the pre‐COVID‐19 period, the period between September 2020 and December 2021 was associated with a decrease of at least one reactive or indeterminate screening test for TTI (OR: 0.753, 95% CI: 0.665–0.854, *p* < 0.001), but did not decrease during the March 2020 to August 2020 period (OR: 0.854, 95% CI: 0.716–1.018, *p* = 0.078). The period between September 2020 and December 2021 was associated with decreased reactive or indeterminate screening tests for syphilis (OR: 0.842, 95% CI: 0.718–0.987, *p* = 0.034), HTLV 1/2 (OR: 0.744, 95% CI: 0.571–0.986, *p* = 0.028) and HBV (OR: 0.840, 95% CI: 0.709–0.997, *p* = 0.046) compared to the pre‐COVID‐19 period. The period between March 2020 and August 2020 was only associated with decreased reactive or indeterminate screening tests for HBV (OR: 0.766, 95% CI: 0.597–0.983, *p* = 0.036) compared to the pre‐COVID‐19 period.

## 4 DISCUSSION

Maintaining a blood supply was a significant issue for most blood centres during the COVID‐19 pandemic.^7^, ^9^, ^10^, ^11^, ^12^ Our study observed a significant decrease in blood donations during the initial stages of the COVID‐19 pandemic, from March 2020 to August 2020. However, after this period, blood donations returned to the levels anticipated by the counterfactual prediction between September 2020 and December 2021. These results reinforce the adverse effect of the COVID‐19 pandemic on blood donations,^7^, ^9^, ^10^, ^11^, ^12^, ^25^ underscoring the effectiveness of the measures implemented by the blood center.^2^, ^21^, ^25^, ^26^, ^27^, ^28^ Meanwhile, a significant decline was observed in the number of individuals with at least one reactive or indeterminate screening test for TTIs, particularly for syphilis, HTLV 1/2, and HBV, in the post‐COVID‐19 onset period compared to the pre‐pandemic period. This finding may be attributed to shifts in blood donor characteristics and behaviours influenced by COVID‐19 prevention and control measures, such as social distancing, which may have also contributed to reducing exposure to TTIs.^13^, ^15^, ^29^ Additionally, there may be considerations regarding potential factors that may influence serological reactivity, such as vaccinations and SARS‐CoV‐2 infection. However, this assessment was limited due to significant differences in the number of individuals who returned for confirmatory testing across the studied periods.^29^, ^30^, ^31^

Even before the COVID‐19 pandemic, maintaining an optimal level of blood stocks was a significant challenge for many blood centres, particularly in middle‐ and low‐income countries, which often faced donation shortages or declines.^20^, ^32^, ^33^ This situation worsened with the COVID‐19 pandemic, resulting in a dramatic decrease in blood donations, a phenomenon also described during previous infectious disease outbreaks, such as the H1N1 pandemic.^23^, ^27^, ^34^, ^35^, ^36^, ^37^ The substantial reduction in blood donations during the COVID‐19 pandemic can be superficially attributed to the fear of becoming infected by blood donors and the restriction of social mobility due to lockdown measures.^21^, ^24^, ^25^, ^28^, ^38^ Indeed, a study in China during the early stage of the COVID‐19 pandemic highlighted that the fear of infection was a key reason for hesitation to donate blood, with nearly 80% of people reporting fear of becoming infected during the donation process.^24^ However, the relationship between beliefs associated with COVID‐19 and blood donation intentions and behaviour is complex and extends beyond simple fear or perceived infection risk.^21^, ^28^, ^38^ In India, a survey including 1066 participants found that more than half (55%) reported experiencing one or more barriers during the COVID‐19 pandemic that hindered blood donation. The most common barriers included the fear of transmitting infection to their families (55.4%) and a lack of confidence in the safety measures implemented by healthcare services (55.4%). Additionally, in a smaller proportion, the fear of contracting COVID‐19 was reported by 26.7% of respondents.^21^

In our study, by September 2020, a few months after the COVID‐19 onset in Brazil in March 2020, blood donations had returned to the levels foreseen by the time‐series model. Notably, this occurred despite ineffective treatments and available COVID‐19 vaccines. This recovery was likely driven by measures implemented by the blood centre, such as donation scheduling, media campaigns, and effective public communication. While the pandemic was beyond the direct control of blood centres, reinforcing the individual motivations for blood donation was crucial in overcoming its challenges. Despite the fear and uncertainty surrounding COVID‐19, factors such as altruism, social approval, coping mechanisms, self‐efficacy, and trust played a key role in sustaining donation intent.^27^, ^28^, ^34^ A well‐structured communication strategy was essential to building trust, emphasising the minimal risk of infection through donation, and demonstrating the commitment of blood centres to donor safety. Media campaigns showcasing operational changes—such as online appointments, thermal screening, mask mandates, rigorous sanitation, and social distancing—helped reassure the public and enhance donor confidence, ultimately strengthening their self‐efficacy.^5^, ^21^, ^25^, ^26^, ^28^, ^39^, ^40^, ^41^

The overall frequency of at least one reactive screening test for TTI (1.2%) in our study was below what was reported among blood centres in Brazil (3.1%).^42^ Our study also revealed a compelling decrease in seroprevalence of TTIs during the COVID‐19 pandemic. This result is particularly noteworthy, especially considering that studies in Brazil indicated a concerning trend for increasing TTIs among blood donors just before the COVID‐19 pandemic.^43^ Similarly, a study in the United States observed a temporary decline in both blood donations and reactive serological tests for certain TTIs after the onset of the pandemic.^13^ Conversely, a study conducted in India found an increase in reactive serological tests for at least one TTI (almost 50% in 2020 and 2021 compared to 2019), with a significant rise in reactivity for syphilis, HBV, and HCV.^22^ This study found the highest prevalence of reactive serological tests among replacement blood donors, suggesting that the increased TTI prevalence may be linked to the higher proportion of replacement donations compared to voluntary donors,^22^ who are widely recognised as the foundation of a safe blood supply.^6^, ^44^, ^45^ However, donor behaviour is complex. In the United States, for example, a decline in first‐time donors was compensated by an increase in donations per donor, potentially contributing to the reduction in HCV seroprevalence. Additionally, there were more donations from female donors, those aged 40 and older. Yet, changes in donor demographics and characteristics did not appear to influence the observed trends in HCV, as reactive test rates declined across most demographic groups.^13^ Despite our study not explicitly quantifying the volume of first‐time and replacement blood donors, according to Brazilian governmental data, replacement blood donations represented 19.8% of all blood donations in the FD in 2021 and 22.9% in 2022.^42^ One possible explanation for reducing seroprevalence for TTIs in our study is the social distancing measures, including lockdowns, implemented during the COVID‐19 pandemic. These restrictions limited social interactions and, consequently, reduced casual sexual encounters.^29^

Our study has some limitations. First, there is a lack of detailed information regarding blood donors, including their motivations for donation, whether they are first‐time or repeat donors, the number of donations per donor, socioeconomic status, and level of education. This is particularly important as the risk of TTIs could potentially increase if a higher proportion of new donors were required during the COVID‐19 pandemic. Thus, acknowledging that other factors not considered in our study could have influenced the group outcomes is essential. Second, the study focused on product discard rather than donor deferral. Third, we did not evaluate the total blood supply during the COVID‐19 pandemic. Despite the decrease in blood collection during the initial stage, there was also a decrease in the number of transfusions due to the postponement of elective surgeries and patient hospitalisations for various reasons, which potentially mitigates the negative impact of reduced blood collections or may even have led to an increase in blood unit discarding rates.^46^ Fourthly, differences in the number of individuals who returned for confirmatory testing across the studied periods limited the evaluation of confirmatory test results. Finally, regarding the causal impact analysis, the impact of COVID‐19 on blood donations involved the combination of three elements: a regression component that relates the outcome of the COVID‐19 period to the outcomes on counterfactual prevision, a time‐series component capturing temporal patterns in the data, and an error element considering any unpredicted variation. Despite its strengths, there are limitations to using the CIA. Specifically, the underlying time‐series model typically entails numerous unknown parameters, necessitating substantial data for accurate estimation, including counterfactual prediction. Regarding these facts, our study included 205 965 blood donations, providing a robust dataset. Further, the performance of the CIA may be affected if the outcome is susceptible to measurement errors. However, this fact does not apply to the outcomes evaluated in our study.^47^, ^48^

## 5 CONCLUSION

Our study revealed the detrimental impact of the COVID‐19 pandemic on blood services, marked by a substantial decrease in blood donations soon after its onset in Brazil. However, within a few months, the donation levels had returned to those projected by the time‐series model despite the significant toll of the pandemic in terms of fatalities in Brazil and the absence of effective treatments and available COVID‐19 vaccines. The measures implemented by the blood centre to navigate the challenges posed by COVID‐19 likely played a pivotal role in this recovery of blood donations, encompassing a clear and consistent communication strategy to build trust among blood donors and reinforcing the individual characteristics that motivate blood donation. An intriguing finding in our study was the decrease in the seroprevalence of TTIs during the COVID‐19 pandemic. This phenomenon may be associated with shifts in blood donor attitudes influenced by preventive measures for COVID‐19, such as social distancing and lockdowns. Subsequent research is warranted to delve into the behavioural patterns of blood donations during pandemics and infectious disease outbreaks.

## AUTHOR CONTRIBUTIONS

Laiane da Silva Santos and Flávio Ferreira Pontes Amorim designed the study. All authors contributed to study conduct, data processing, data analysis, data review, and data interpretation. Laiane da Silva Santos, Sérgio Eduardo Soares Fernandes, and Flávio Ferreira Pontes Amorim were responsible for the statistical analysis. All authors wrote and reviewed the manuscript.

## FUNDING INFORMATION

The authors received no financial support for the research, authorship, and/or publication of this article.

## CONFLICT OF INTEREST STATEMENT

The authors declare no conflicts of interest.

## PATIENT CONSENT STATEMENT

The study was approved by the institutional review board with a waiver of informed consent as the study had no specific intervention and used only anonymised retrospective medical record data that generated results in an aggregate manner that did not allow identification of participants.

## Supporting information


**Supplementary Table S1.** Causal impact analysis of the COVID‐19 pandemic on blood donations in the FD public health system adjusted to the FD population using data from March 2018 to December 2021.
**Supplementary Table S2.** Cochran‐Armitage test for trend in proportions of blood discards resulting from a single agent and mixed agents of transfusion transmitted infections (TTIs) by the period of blood donation (*n* = 205 965).

## Data Availability

The data that support the findings of this study are available from the corresponding author upon reasonable request.
