# Supplementary material for: COVID‐19 pandemic impact on blood donations and discards from screening tests for transfusion‐transmitted infections in a Brazil Brazilian metropolitan area
Source: Transfus Med. 2025 Jul 7;35(6):570–81. doi: 10.1111/tme.13159 (PMC12722195; doi:10.1111/tme.13159)
Supplement: Supplementary file 1 — Supplementary Table S1. Causal impact analysis of the COVID‐19 pandemic on blood donations in the FD public health system adjusted to the FD population using data from March 2018 to December 2021. Supplementary Table S2. Cochran‐Armitage test for trend in proportions of blood discards resulting from a single agent and mixed agents of transfusion transmitted infections (TTIs) by the period of blood donation (n = 205 965). [file TME-35-570-s001.docx]

**Supplementary File**

**Supplementary Table S1.** Causal impact analysis of the COVID-19 pandemic on blood donations in the FD public health system adjusted to the FD population using data from March 2018 to December 2021

| Study period* | Total blood donations | Expected blood donations with the COVID-19 pandemic had not occurred  (95% CI) | Absolute effect  (95% CI) | Relative effect, %  (95% CI) |
| --- | --- | --- | --- | --- |
| Mar 2020–Aug 2020 | 23,764 | 26,716  (25,119 to 28,391) | -2,952  (-4.627.0 to -1.355) | -11.0  (-16.3 to -5.4) |
| Sep 2020–Dec 2021 | 69,381 | 68,142  (62,638 to 73,814) | 1,239  (-4,433 to 6,743) | 1.8  (-6.0 to 10.8) |
| Mar 2020–Dec 2021 | 93,145 | 96,817  (89,343 to 105,014) | -3,672  (-11,869 to 3,802) | -3.8  (-11.3 to 4.2) |

95% CI: 95% confidence interval.

*Study period: From the first epidemiological week of the first month to the last epidemiological week of the last month of the analyzed period

**Supplementary Table S2.** Cochran-Armitage test for trend in proportions of blood discards resulting from a single agent and mixed agents of transfusion transmitted infections (TTIs) by the period of blood donation (n = 205,965)

|  | Single agent  (reactive or indeterminate screening test for only one TTI) | | Mixed agent (reactive or indeterminate screening test for more than one TTI) | | No reactive or indeterminate screening test for TTI | | p-value |
| --- | --- | --- | --- | --- | --- | --- | --- |
|  | n | % | n | % | n | % |  |
| Jan 2018–Feb 2020 | 1,449 | 1.3 | 59 | 0.1 | 111,188 | 98.7 | < 0.001 |
| Mar 2020–Aug 2020 | 277 | 1.1 | 14 | 0.1 | 24,402 | 98.8 |  |
| Sep 2020–Dec 2021 | 652 | 1.0 | 34 | < 0.1 | 67,890 | 99.0 |  |
